# Photocatalytic Gold Recovery from Industrial Gold Plating Effluent by ZnO Nanoparticles: Optimum Condition and Possible Applications

**DOI:** 10.1021/acsomega.3c07336

**Published:** 2023-11-13

**Authors:** Auttawit Thoumrungroj, Suchalee Wongtongprapun, Soontorn Tuntithavornwat, Chonticha Saisawang, Satjaporn Sangkhanak, Panuwat Wongyongnoi, Karn Serivalsatit, Mali Hunsom

**Affiliations:** † Department of Chemical Engineering, Faculty of Engineering, Mahidol University, Nakhon Pathom 73170, Thailand; ‡ Advanced Microfabrication and Biomaterial for Organ-on-chip Research Unit (AMBiO), Faculty of Engineering, Mahidol University, Nakhon Pathom 73170, Thailand; § Institute of Molecular Biosciences, Mahidol University, Nakhon Pathom 73170, Thailand; ∥ Department of Chemical Technology, Faculty of Science, Chulalongkorn University, Bangkok 10330, Thailand; ⊥ Department of Materials Science, Faculty of Science, Chulalongkorn University, Bangkok 10330, Thailand; # Royal Society of Thailand (AFRST), Bangkok 10300, Thailand

## Abstract

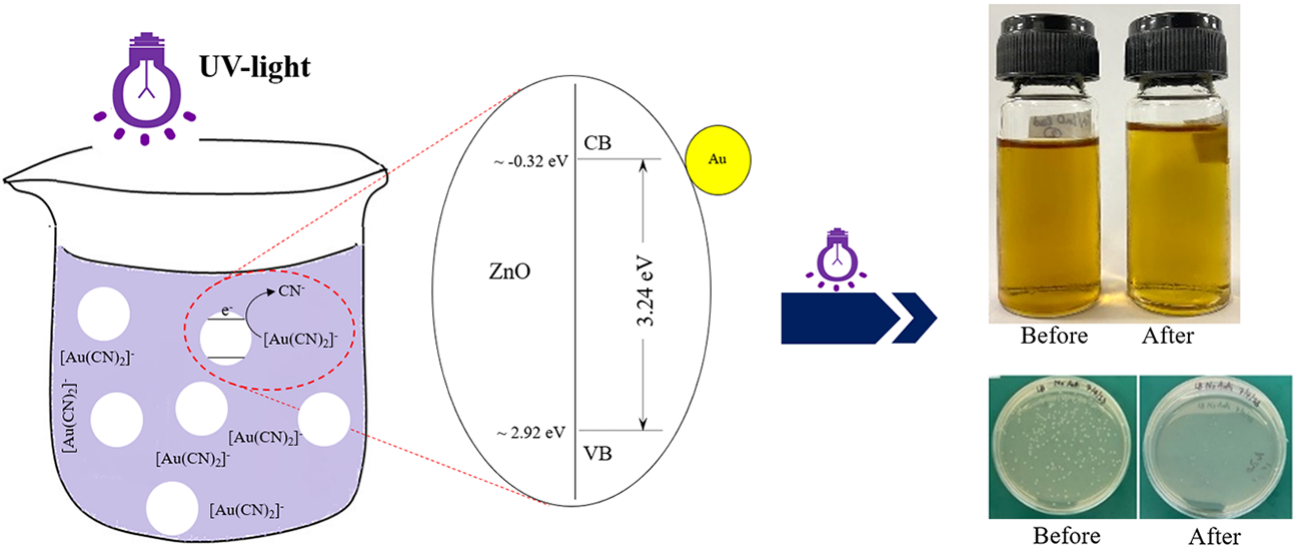

The comparative study of photocatalytic gold recovery from cyanide-based gold plating solution was explored via commercial and hydrothermally synthesized ZnO nanoparticles (NPs). The effects of hydrothermal temperatures on the properties and photocatalytic activities of synthesized ZnO NPs were investigated. In addition, the effects of operating parameters including types of hole scavenger, concentrations of the best hole scavenger, the initial pH of wastewater, and photocatalyst dosages were examined. The obtained results demonstrated that the commercial ZnO NPs exhibited a higher photocatalytic activity for gold recovery than that of the synthesized ones owing to their good crystal quality and the presence of non-lattice zinc ions and appropriate non-lattice oxygen ions. Via the commercial ZnO NPs, the gold ions were almost completely recovered from the cyanide-based gold plating effluent within 7 h at an initial pH of 11.0 in the presence of 10 vol % C_2_H_5_OH and 1.0 g/L of photocatalyst loading with a pseudo-first-order rate constant of 0.2637 h^–1^. Finally, the resultant gold-decorated ZnO NPs exhibited a higher photocatalytic property for color reduction from industrial wastewater and antibacterial activity than that of fresh ZnO NPs. The results obtained in this study possess benefits and pave the way for waste remediation and management for the plating industries.

## 1 Introduction

Gold is one of the most widely used materials with extensive applications in printed circuit boards for fabricating electrical contacts and wiring pads owing to its exceptional properties, including high electrical conductivity, reliability, and corrosion resistance.^1,2^ The production of gold-deposited specimens involves two predominant techniques: noncyanide-based and cyanide-based gold plating.^1^ Notably, the cyanide-based gold plating prevails over the noncyanide-based plating because it provides a stable gold film characterized by aesthetically pleasing attributes and superior physicochemical properties.^1,3^ Nevertheless, the cyanide-based gold plating process still faces some limitations as it discharges the process effluent containing gold species in the form of gold-cyanide complexes.^3,4^ The release of excess gold ions results in economic loss, while the discharge of free cyanide ions induces substantial damage to both human health and the environment.

Currently, various methodologies have been implemented to recover gold concurrently with the conversion of cyanide species into a more stable form. These techniques include chemical oxidation,^5^ solvent extraction,^6,7^ and adsorption.^8−12^ Another promising process that offers the dual benefits of gold recovery and mitigation of cyanide toxicity is the photocatalytic process.^13^ This process is distinguished by its straightforward operation, cost-effectiveness, wide applicability, and environmental friendliness.^14−16^ The photocatalytic process involves a multistep chemical reaction facilitated by the presence of appropriately irradiated light. Upon absorption of photons with adequate photon energy, electrons in the photocatalyst will be excited from the valence band (VB) to the conduction band (CB), leaving the photogenerated holes (h^+^). The ensuing electrons and holes exhibit the reducing and oxidizing properties, respectively.^17^ As described by van Grieken et al.,^13^ the gold-cyanide complexes ([Au(CN)_2_]^−^) within effluent can react with the photogenerated electrons to yield a deposition of metallic gold on the surface of the utilized photocatalyst, concomitant with the release of free cyanide ions (CN^–^) (eq 1). Simultaneously, the added basic compounds can react with the photogenerated holes to form the hydroxyl radicals (OH^•^) (eq 2), which subsequently react with the free cyanide ions to form the more stable cyanate (OCN^–^) species (eq 3).^13^
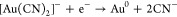
1
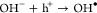
2
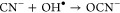
3

However, the utilization of gold-cyanide complexes faces a challenge due to their low reduction potential (−0.57 to −0.60 V/NHE^13,18^), limiting their capability with photocatalysts possessing extremely low negative VB values. Among the well-established photocatalysts, titanium dioxide (TiO_2_) or TiO_2_-based nanoparticles (NPs) have gained prominence as frequently utilized photocatalysts for gold recovery as well as metal removal/recovery due to their favorable textural property, robust photochemical and thermal stability, and environmental friendliness.^4,13,15,16,19−23^

Currently, zinc oxide (ZnO) has emerged as a promising alternative to TiO_2_ driven by its cost-effectiveness, nontoxicity, high electron mobility, excellent electronic and optical properties, straightforward nanostructure modulation, and ease of reconfiguration.^24,25^ Besides, despite sharing a comparable bandgap energy with TiO_2_, it exhibits a high efficiency to absorb a substantial portion of the solar spectrum.^26^ Based on literature, ZnO NPs have been applied in air purification, dye degradation, antibacterial materials, and hydrogen production.^27−29^ Notably, several metal-doped ZnO NPs were applied for photocatalytic applications such as Au/ZnO,^30−32^ Pt/ZnO,^33,34^ Ag/ZnO,^35^ and Pd/ZnO.^36^ However, the utilization of ZnO or ZnO-based NPs for photocatalytic metal ion removal and recovery has been relatively scarce. For instance, the ZnO NPs synthesized by the solution combustion method exhibited exceptional efficiency in gold ion recovery from wastewater containing K, P, Au, Na, Ni, Cu, and Zn ions owing to their appropriate VB edge.^4^ Furthermore, the ZnO NPs synthesized using the sonochemical method demonstrated distinct characteristics and reduction rates of Cr^6+^ depending on the employed solvents.^37^ With an ethanol solvent, the resultant ZnO NPs exhibited approximately twice the Cr^6+^ reduction efficiency compared to that of the commercial ZnO NPs.

The ZnO NPs synthesized by a solid precipitation technique revealed a high photocatalytic activity to remove copper (Cu^2+^), silver (Ag^+^), lead (Pb^2+^), and chromium (Cr^6+^) but relatively poor activity to remove manganese (Mn^2+^), cadmium (Cd^2+^), and nickel (Ni^2+^) depending on the light source and reduction mechanism.^38^ Impressively, high-ratio (100) plane-exposed ZnO nanosheets, synthesized by a hydrothermal method, provided a simultaneous Cr^6+^ reduction and Cr^3+^ adsorption rate over 90% at 120 min under the simulated sunlight and neutral conditions,^39^ attributed to the synergistic effect of Zn- and O-site on the (100) plane.^39^

In this work, the ZnO NPs were synthesized by a facile hydrothermal method to recover gold from an actual cyanide-gold plating bath solution. The investigation delved into the impact of synthesis temperatures on both the morphology and photocatalytic activity of synthesized ZnO NPs in comparison with the commercial ones. Moreover, the potential applications of the resultant gold-decorated ZnO NPs as photocatalysts were extended to color reduction and antibacterial applications. Notably, the utilization of ZnO NPs for gold recovery from real plating wastewater remains largely unexplored. As a result, the outcomes of this research may pave the path toward effective waste remediation and management strategies.

## 2 Results and Discussion

### 2.1 Morphology and Characteristics of ZnO NPs

**Figure 1:**
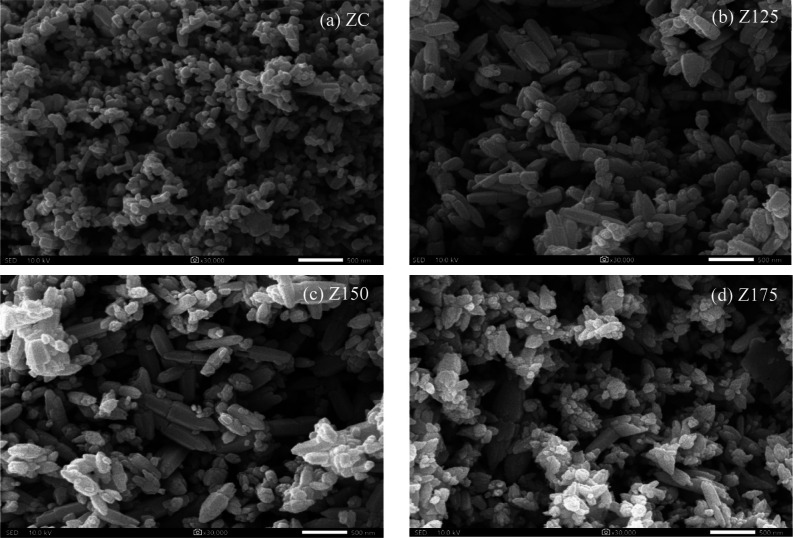
Representative SEM images of (a) ZC, (b) Z125, (b) Z150, and (d) Z175 NPs.

The external features of both commercial and synthesized ZnO NPs were first examined by scanning electron microscopy (SEM) analysis. As depicted in Figure 1, the ZC sample exhibited a finely spherical shape (Figure 1a). Conversely, the synthesized ZnO NPs, specifically the Z125 samples, exhibited rod-like nanostructures with distinct sharp edges resembling the tip of a pencil (Figure 1b). As for the Z150 and Z175 samples, they retained the rod-like structure with sharp tips, albeit with the agglomerated small fragments like a hierarchical flower-like structure (Figure 1c,d). The prevalence of the hierarchy flower-like configuration increased with rising hydrothermal temperatures, underscoring the significant influence of hydrothermal temperature on the nanostructure of ZnO NPs.

**Figure 2:**
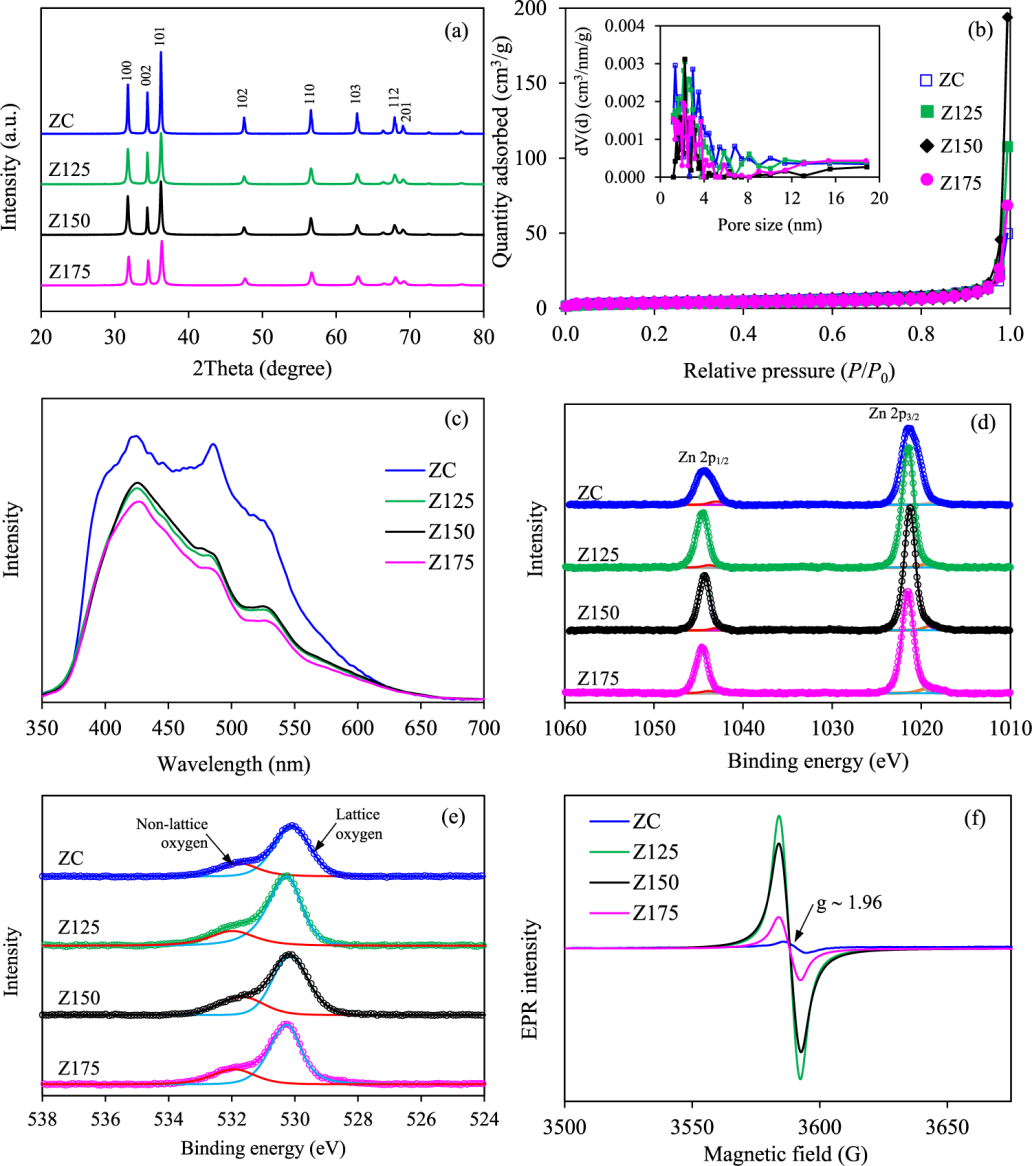
Representative (a) XRD diffractogram, (b) N_2_ adsorption/desorption isotherms, (c) PL spectra, (d) Zn 2p HR XPS spectra, (e) O 1s HR XPS spectra, and (f) EPR spectra of commercial and synthesized ZnO NPs.

Regarding the crystallite structures of all ZnO NPs, the ZC sample displayed the X-ray diffraction (XRD) peaks characteristic of a wurtzite hexagonal structure (JCPDS file no. 36-1451).^24,40,41^ Specifically, diffraction peaks were observed at 2θ values of 31.8, 34.4, 36.3, 47.5, 56.6, 62.8, 67.9, and 69.08°, corresponding to the (100), (002), (101), (102), (110), (103), (112), and (201) crystal planes, respectively (Figure 2a). Remarkably, all synthesized ZnO NPs demonstrated identical characteristic peaks of hexagonal ZnO NPs without additional peaks or shifts in diffraction angles when compared to their commercial counterparts. This observation suggested that the utilized hydrothermal process gave high-purity ZnO NPs in the absence of structural stress development. Nevertheless, distinctions in diffraction peaks between the commercial and synthesized ZnO NPs were evident in terms of peak sharpness. Notably, the ZC sample exhibited greater peak sharpness compared to that of all synthesized ZnO NPs. That is, the intensity of the (101) peak in ZC exceeded that of Z125, Z150, and Z175 by approximately 1.59, 1.52, and 1.83 times, respectively. This discrepancy underscored a superior crystallinity of ZC compared to that of synthesized counterparts. Differential crystal quality is likely to play a crucial role in the photocatalytic activity of ZnO NPs for gold recovery from the cyanide-based gold plating effluent. The crystallite size of all ZnO NPs was calculated according to the Debye–Scherrer eq (eq 4)^42^ using the (101) crystal plane. As summarized in Table 1, the calculated crystallite size of all synthesized ZnO NPs was lower than that of the ZC sample.
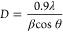
4

**Table 1:** Properties of Commercial and Synthesized ZnO NPs

catalysts	crystallite size (nm)^a^	textural property			bandgap energy (eV)
		surface area (m^2^/g)	mean pore size (nm)	mean pore volume (cm^3^/g)	
ZC	41.58	14.2	1.35	0.077	3.24
Z125	28.63	16.9	2.25	0.169	3.21
Z150	29.11	13.9	2.25	0.299	3.21
Z175	24.96	11.5	2.12	0.104	3.22

a Calculated from XRD analysis using the Debye–Scherrer equation at the crystal plane of (101).

where *D* is the crystallite size (nm), λ is the wavelength of the X-ray radiation (0.154178 nm), β is the full width at half-maximum intensity of the peak, and θ is the diffraction angle.

The textural characteristics of commercial and synthesized ZnO NPs were assessed through a N_2_ adsorption/desorption analysis. As depicted in Figure 2b, both commercial and synthesized ZnO NPs exhibited the type IV isotherm according to the IUPAC classification, indicating the presence of abundant mesoporous structures.^43,44^ Akin hysteresis loops were observed across all samples, demonstrating pronounced N_2_ adsorption/desorption at a relative pressure (*P*/*P*_0_) of 0.95–0.97. This phenomenon suggests a consistent regularity among the samples coupled with a broad pore size distribution. The ZC sample showcased a BET surface area of 14.2 m^2^/g, accompanied by small mean pore sizes and pore volumes (Table 1). The Z125 NPs exhibited slightly higher surface areas compared with those of the commercial one. Increasing the synthesized hydrothermal temperature decreased the textural property considered in terms of surface area, mean pore size, and mean pore volume.

The photoluminescence (PL) characteristics of all samples were subsequently evaluated by using an excitation wavelength of 286 nm. Typically, PL emissions within the UV spectrum often reflect crystal quality, while those within the visible light spectrum signify structural defects.^45,46^ A heightened PL emission intensity ratio between the UV and visible regions indicates reduced defect concentration and enhanced crystal quality.^46^ Besides, a high PL intensity also indicates a fast recombination rate of photogenerated charges or, alternatively, denotes the diminished electron–hole pair separation efficiency.^41,47^ As demonstrated in Figure 2c, all samples displayed three prominent emission bands centered at ∼420, ∼484, and ∼530 nm. These correspond to the electron transitions from Zn interstitials (Zn_i_) to VB, ionized oxygen vacancy (O_v_) to VB, and oxygen interstitial (O_i_) from CB to O_Zn_ level.^46,48,49^ Remarkably, the ZC sample exhibited more pronounced emission bands compared to those of the synthesized ZnO samples, suggesting superior crystal quality and a higher number of defects,^25,45,46^ along with the marginally accelerated recombination rate of generated charges. On the other hand, the PL spectra of all synthesized ZnO samples possessed comparable PL spectra, suggesting that the utilized hydrothermal temperatures in the range of 125–175 °C exerted an insignificant influence on the crystal quality and structural defects of the resultant ZnO NPs.

For an in-depth investigation of potential defects within the structure of ZnO NPs, X-ray photoelectron spectroscopy (XPS) analysis was conducted. As depicted in Figure 2d, the high-resolution XPS spectra of ZC displayed discernible peaks corresponding to Zn 2p_3/2_ and Zn 2p_1/2_ at binding energies of 1021.34 and 1044.38 eV, respectively. The binding energy separation of ∼23 eV indicated the presence of zinc in the Zn^2+^ oxidation state.^41,50,51^ Besides, two subpeaks centered at 1018.40 and 1043.04 eV were observed, arising from Zn 2p_3/2_ and 2p_1/2_ associated with non-lattice zinc ions (e.g., Zn_i_, singly charged zinc interstitials (Zn_i_^+^), or doubly charged zinc vacancies (V_Zn_^2–^)).^52^ Similarly, in the case of synthesized ZnO NPs, their XPS spectra exhibited the Zn 2p doublet corresponding to both lattice zinc ions (∼1021 and ∼1044 eV) and non-lattice zinc ions (∼1019 and ∼1043 eV). The high-resolution O 1s XPS spectra of both ZC and all synthesized ZnO NPs exhibited distinctive asymmetric broad peaks maximized at ∼530 eV (Figure 2e), aligned with lattice oxygen.^52^ Furthermore, subpeaks located at 531.74, 531.98, 531.65, and 531.92 eV of ZC, Z125, Z150, and Z175 were respectively observed, corresponding to non-lattice oxygen (oxygen vacancy) ions,^52^ in agreement with the PL analysis. The coexistence of non-lattice zinc and oxygen ions in the structure could potentially play a key role in the photocatalytic reduction of gold from industrial gold plating effluent.

**Figure 3:**
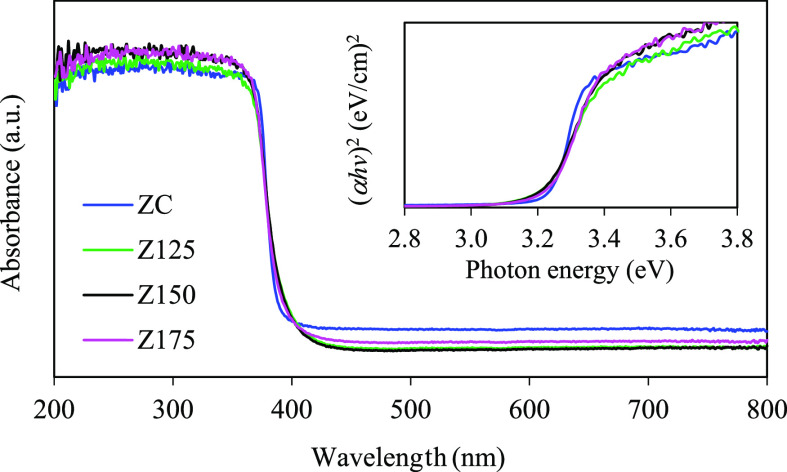
UV–vis absorption spectra of commercial and synthesized ZnO NPs and their Tauc plots.

The intrinsic defects presented in both commercial and synthesized ZnO NPs were additionally explored via an EPR analysis. Typically, the output of the EPR signal at an isotropic *g*-factor of ∼1.96 indicates the presence of unpaired electrons resulting from bulk oxygen vacancies.^30,53−55^ A high strength of the EPR signal indicates a high concentration of bulk oxygen vacancies.^56−58^ As plotted in Figure 2f, all of the synthesized ZnO samples exhibited a stronger EPR signal than that of the ZC one. This observation suggests that all synthesized ZnO NPs contained a higher quantity of bulk oxygen vacancies than their commercial counterpart. The light absorption capability of all ZnO NPs was then analyzed by a UV–vis absorption spectrophotometer. All peaks that appeared in the absorption spectra were attributed to the electron transfer between the VB, CB, or interstage levels.^40^ As shown in Figure 3, both synthesized and commercial ZnO NPs displayed prominent broad peaks at wavelengths below 390 nm, indicating their effective UV light absorption. According to Tauc’s plot (inset of Figure 3), the bandgap energies of ZC, Z125, Z150, and Z175 were determined to be 3.24, 3.21, 3.21, and 3.22 eV, respectively (Table 1). As mentioned elsewhere, the variation of the optical property of ZnO NPs is dictated by various factors such as catalyst morphology, crystallite size, crystallinity, defects, and also impurity contents.^45^

### 2.2 Photocatalytic Activity

**Figure 4:**
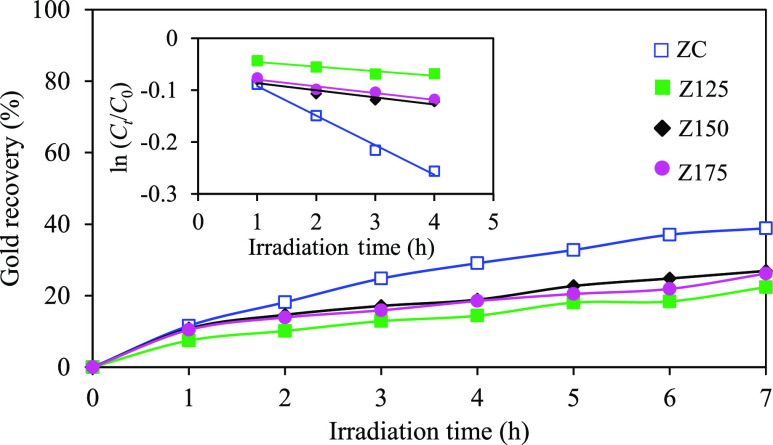
Effect of ZnO NP types on the photocatalytic gold recovery at a light intensity of 4.93 mW/cm^2^, a catalyst loading of 2.0 g/L, and an initial pH of effluent of 6.0. in the presence of 20 vol % C_2_H_5_OH as a hole scavenger.

The investigation into the photocatalytic activity of both commercially available and hydrothermally synthesized ZnO NPs was explored for gold recovery from the cyanide-based gold plating effluent with gold ion concentrations ranging from around 7 to 10 mg/L. The experiments were carried out under a light intensity of 4.93 mW/cm^2^, a catalyst loading of 2.0 g/L, and an initial pH of effluent of 6.0 in the presence of 20 vol % ethanol. As illustrated in Figure 4, the ZC sample exhibited gold recovery percentages of approximately 38.8% within 7 h, which was considerably higher than those of synthesized ZnO NPs of around 1.4–1.7 times. Employing the Langmuir–Hinshelwood model, the pseudo-first-order rate constant for ZC was determined as 0.0569 h^–1^, approximately 6.5, 4.2, and 4.4 times higher than that of Z125, Z150, and Z175, respectively.

Considering the morphological and optical properties of all ZnO NPs, despite the slightly reduced separation efficiency of photogenerated charges and marginally elevated bandgap energy in the ZC sample compared to those of the synthesized ZnO NPs, the former exhibited a superior photocatalytic activity for gold recovery from the cyanide-based gold plating effluent. This suggests that the large fraction of high-quality crystal structures and defective configurations of ZC played a more prominent role on the photocatalytic gold recovery, surpassing the influence of bandgap energy and the rate of electron–hole recombination. Specifically, the quality of the crystal within ZnO NPs can substantially facilitate the efficient movement of generated electrons along the photocatalyst nanostructures,^25,59^ thus promoting the rate of photocatalytic gold recovery. Besides, the presence of suitable defective sites can function as charge-trapping locations^60,61^ or active sites that catalyze the reduction of ionic gold to metallic gold NPs. Although all synthesized ZnO NPs contained a high concentration of oxygen vacancies, they possessed a considerably low photocatalytic performance compared with that of the ZC NPs. This disparity could be attributed to the excessive oxygen vacancies in the synthesized ZnO NPs, which potentially acted as the recombination center,^62,63^ thus exerting a detrimental impact on the photocatalytic activity for gold recovery.

**Figure 5:**
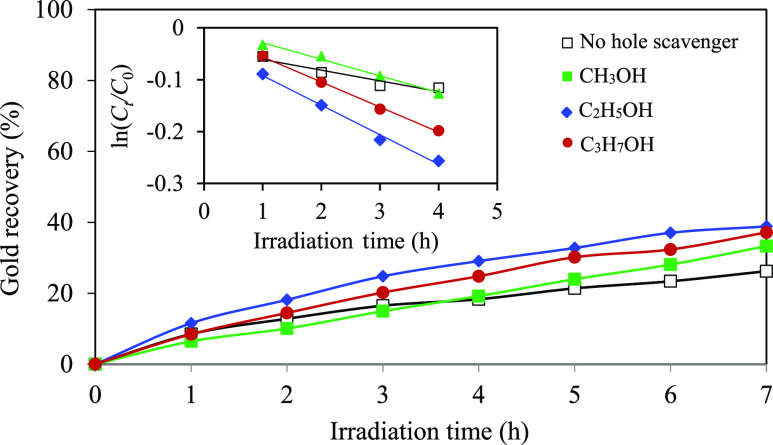
Effect of hole scavenger types on the photocatalytic gold recovery at a light intensity of 4.93 mW/cm^2^, a catalyst loading of 2.0 g/L, and an initial pH of effluent of 6.0.

It is widely recognized that the presence of sacrificial agents can significantly improve the photocatalytic activity by mitigating the rate of electron–hole recombination.^64,65^ In this work, three types of alcohols including CH_3_OH, C_2_H_5_OH, and C_3_H_7_OH were employed as hole scavengers due to their low oxidation potential, rendering them as excellent candidates for hole scavenging.^66^ As depicted in Figure 5, approximately 26.2% of gold was recovered within 7 h in the absence of hole scavengers. However, the presence of CH_3_OH, C_2_H_5_OH, and C_3_H_7_OH promoted the gold recovery efficiency to 33.3, 38.8, and 37.2%, respectively. Based on the Langmuir–Hinshelwood model, the calculated pseudo-first-order rate constants for systems with CH_3_OH, C_2_H_5_OH, and C_3_H_7_OH were 0.0319, 0.0569, and 0.0483 h^–1^, respectively. These values were approximately 1.5, 2.8, and 2.3 times higher than those observed in the absence of a hole scavenger. This indicates that the added hole scavengers can effectively extend the lifetime of the electron–hole pairs. Different rates of photocatalytic gold recovery in the presence of different hole scavengers are probably caused by their different standard oxidation potentials. Chemicals with lower standard oxidation potentials tend to perform efficiently as electron donors or hole scavengers.^66−68^ That is, the standard oxidation potentials of CH_3_OH, C_2_H_5_OH, and C_3_H_7_OH were determined as 0.016, 0.084, and 0.105 V/NHE, respectively.^66^ However, the experimental findings did not follow the oxidation potential trend. Surprisingly, the system employing C_2_H_5_OH exhibited the highest photocatalytic activity, while the CH_3_OH system exhibited the lowest photocatalytic activity. As mentioned previously,^66^ the CH_3_OH oxidation via photogenerated holes can be expressed by eqs 5–8. The ensuing H_2_C*OH radicals, stemming from eq 5, can then react with adsorbed OH(a) and holes with the use of electrons to form H_2_ and HCHO (eq 6). Subsequently, the formed HCHO species can further react with photogenerated holes and electrons, yielding H_2_ and adsorbed CO(a) as products (eq 7). Although the photogenerated holes are consumed within this series of reactions, thus minimizing the electron–hole recombination rate, the photogenerated e^–^ are also utilized. This competitive utilization of the photogenerated electrons may consequently attenuate the photocatalytic rate of gold recovery.
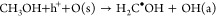
5
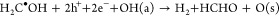
6
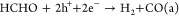
7
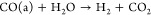
8

In the presence of C_2_H_5_OH, the hole-trapping mechanism of C_2_H_5_OH was already proposed via the use of the TiO_2_ semiconductor as demonstrated by reactions (R9) to (R11).^69^ Notably, this hole entrapment reaction of C_2_H_5_OH is not involved with photogenerated electrons, ensuring that almost all generated electrons remain available for participation in the photoreduction of gold ions to metallic gold (eq 1), thus enhancing the photocatalytic performance. Moreover, intermediate species resulting from this process can react with adsorbed oxygen (O(s)) (eqs 9 and 10), contributing to the reduction of soluble oxygen in the solution.^70,71^ This reduction alleviates the competitive reduction reaction between dissolved oxygen and gold ions.^13^ Additionally, the generated CH_3_C^•^HO(a) species can readily react with the surplus photogenerated holes (eq 11), thus effectively suppressing the rate of electron–hole recombination. Although the addition of C_2_H_5_OH as a hole scavenger may affect the CN^–^ oxidation due to the competitive reactions with the photogenerated holes as described in eqs 2 and 3 and 9–11, its inclusion exhibited a favorable impact on the overall photocatalytic activity for gold recovery (Figure 5).
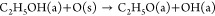
9
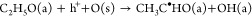
10
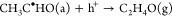
11

**Figure 6:**
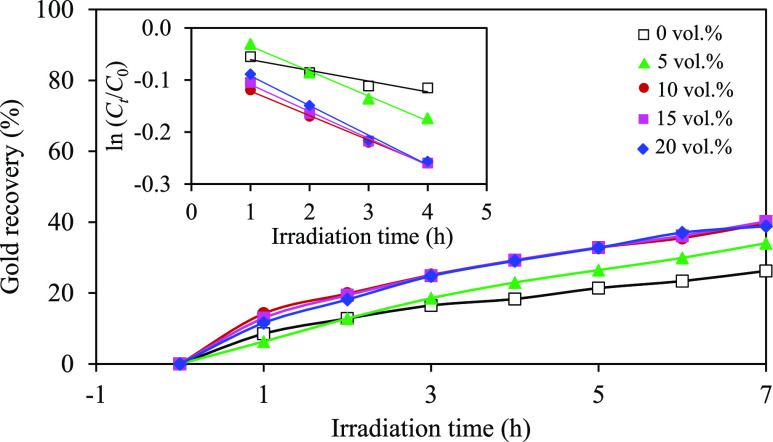
Effect of the C_2_H_5_OH concentration on the photocatalytic gold recovery at a light intensity of 4.93 mW/cm^2^, a catalyst loading of 2.0 g/L, and an initial pH of effluent of 6.0.

To optimize the hole scavenger quantity, the effect of varying C_2_H_5_OH concentrations on the photocatalytic gold recovery was systematically explored using the ZC sample. This experiment was conducted under a light intensity of 4.93 mW/cm^2^, a catalyst loading of 2.0 g/L, and an initial pH of 6.0 in the presence of 0–20 vol % C_2_H_5_OH. In the absence of C_2_H_5_OH, approximately 26.2% of gold ions were recovered within 7 h (Figure 6). Subsequent increments in C_2_H_5_OH concentration from 0 to 10 vol % led to an increased gold recovery percentage, reaching 39.6%. However, further increasing the C_2_H_5_OH concentration to 20 vol % did not result in additional enhancement of gold recovery. Via the Langmuir–Hinshelwood model, the pseudo-first-order rate constants for the system with C_2_H_5_OH concentrations of 0, 5, 10, 15, and 20 vol % were determined as 0.0206, 0.0478, 0.0470, 0.0517, and 0.0569 h^–1^, respectively. The relatively poor photocatalytic gold recovery at lower C_2_H_5_OH concentrations (<10 vol %) could be attributed to the limitation of hole scavengers to effectively capture photogenerated holes.^72^ Conversely, at high C_2_H_5_OH concentrations (>10 vol %), a large quantity of C_2_H_5_OH can adsorb onto the photocatalyst surface without substantial contribution to the reaction, probably due to the fixation of light intensity and other operating parameters. This suggests that, within the experimental framework, a critical balance of hole scavenger concentrations appears most effective for optimal photocatalytic gold recovery, 10 vol % of hole scavenger or C_2_H_5_OH in this context.

**Figure 7:**
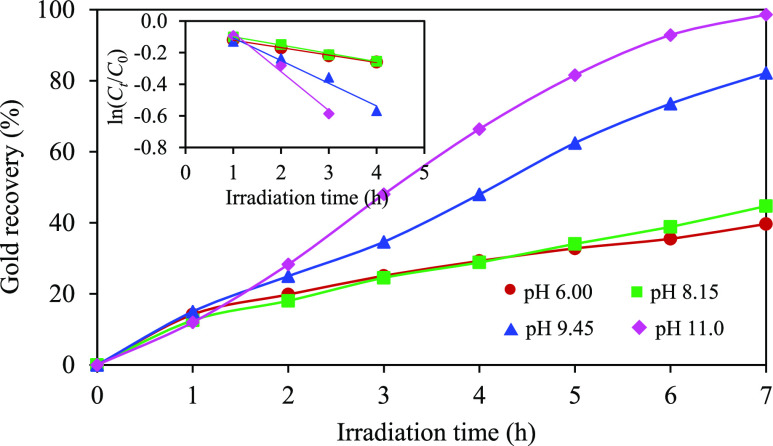
Effect of the initial pH of effluent on the photocatalytic gold recovery at a light intensity of 4.93 mW/cm^2^ and a catalyst loading of 2.0 g/L in the presence of 10 vol % C_2_H_5_OH.

Figure 7 illustrates the variation in photocatalytic gold recovery over a specific time frame, employing the ZC photocatalyst under diverse initial pH values of the effluent, ranging from 6.0 to 11.0. This experimental investigation maintained a constant light intensity of 4.93 mW/cm^2^, a catalyst loading of 2.0 g/L, and a C_2_H_5_OH concentration of 10 vol %. Notably, increasing the initial pH of the effluent from 6.0 to 11.0 resulted in a progressive increase in the percentage of gold recovery, scaling from 39.7 to 98.6% over a 7 h irradiation period. According to the Langmuir–Hinshelwood model, the pseudo-first-order rate constant increased from 0.0470 to 0.2461 h^–1^. Actually, there are many possible reasons that may affect the photocatalytic performance of gold recovery under different pH conditions. The first one might be attributed to the relationship between the surface charges and the forms of gold cyanide complexes. One potential determinant pertains to the interplay between surface charges and the forms of gold cyanide complexes. Generally, semiconductors demonstrate positive surface charges when the solution pH falls below the PZC value, transitioning to negative surface charges when the solution pH surpasses the values.^73,74^ However, gold cyanide complexes usually exhibit the stable form [Au(CN)_2_]^−^ across a wide pH range at 25 °C and 1 atm.^75^ Consequently, the repulsive interaction stemming from the negative surface charge of the ZC sample and the negative charges of the gold cyanide complexes can be negligible in this context. Another contributing factor may be the presence of protons (H^+^) and/or hydroxide ions (OH^–^) within the solutions. Elevated H^+^ quantity in acidic solution could potentially initiate competitive reduction reactions with [Au(CN)_2_]^−^, thus reducing the photocatalytic activity of gold recovery. Alternatively, in the presence of high OH^–^ contents, these entities can readily react with photogenerated holes, giving rise to the formation of OH^•^ at VB.^13^ This phenomenon, in turn, effectively mitigates the rate of electron–hole recombination, thereby facilitating improved photocatalytic gold recovery.

**Figure 8:**
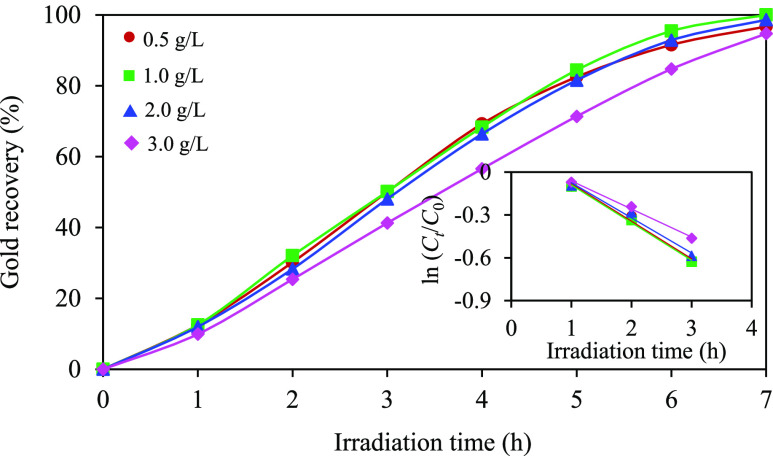
Effect of photocatalyst loading on the photocatalytic gold recovery at a light intensity of 4.93 mW/cm^2^ and an initial pH of effluent of 11.0 in the presence of 10 vol % C_2_H_5_OH.

In consideration of economic feasibility, the content of the utilized ZC sample needs to be minimized. Figure 8 illustrates the effect of photocatalyst loading on the efficiency of photocatalytic gold recovery, utilizing a consistent light intensity of 4.93 mW/cm^2^, an initial wastewater pH of 11.0, and 10 vol % C_2_H_5_OH. It can be seen that the photocatalytic system employing photocatalyst loadings of 0.5, 1.0, and 2.0 g/L exhibited comparable gold recovery percentages, accompanied by pseudo-first-order rate constants of 0.2629, 0.2637, and 0.2461 h^–1^, respectively. Conversely, raising the photocatalyst loading to 3.0 g/L decelerated the percentage of gold recovery, accompanied by a diminished pseudo-first-order rate constant of 0.1963 h^–1^. A poor photocatalytic activity in the presence of extremely high photocatalyst loading usually mirrors the trend observed in other photocatalytic systems.^76−79^ The diminished performance at extremely high photocatalyst loadings can be attributed to several factors. First, such conditions can induce a saturation of adsorbed photons due to continuous irradiation, impeding the efficiency of the photocatalytic process. Additionally, an excessive loading can lead to light blockage or shading behavior,^79,80^ thus limiting the light penetration to the photocatalyst surface and consequently restraining its photocatalytic activity. Hence, maintaining a balanced photocatalyst loading is pivotal to achieving optimal photocatalytic performance.

### 2.3 Possible Applications of Resultant Gold-Decorated ZnO NPs

**Figure 9:**
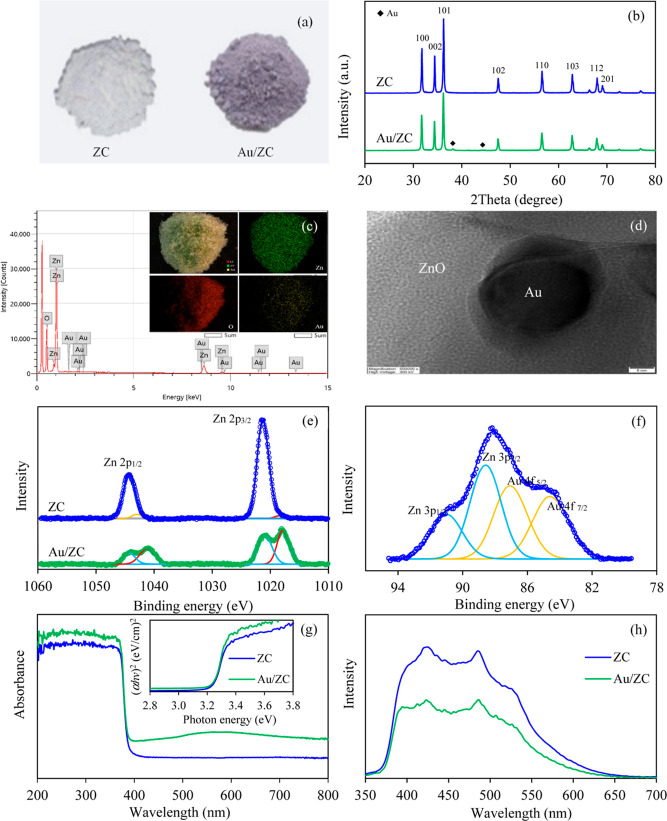
Representative (a) external feature, (b) XRD pattern, (c) SEM–EDX image, (d) TEM image, (e) Zn 2p XPS spectra, (f) Au 4f XPS spectra, (g) UV–vis absorption spectra and Tauc plots, and (h) PL spectra of the Au/ZC sample.

As mentioned in prior discussion, gold-decorated commercial ZnO NPs (Au/ZC) generally exhibit various intrinsic properties that render them valuable for a wide variety of photocatalytic applications, such as dye degradation,^31,81−84^ H_2_ production from water splitting,^85−87^ and chemical production.^88^Figure 9a presents digital pictures of the resulting Au/ZC sample together with the original ZC sample. In contrast to the pristine ZC sample, Au/ZC exhibited a discernible purple-gray color due to the localized surface plasmon resonance generated by the decorated Au NPs. Regarding the crystallite structure, both samples exhibited the XRD peaks of a wurtzite hexagonal structure. The diffraction peaks correspond to 2θ values of 31.8, 34.4, 36.3, 47.5, 56.6, 62.8, 67.9, and 69.08°, aligning with the crystal planes of (100), (002), (101), (102), (110), (103), (112), and (201), respectively (Figure 9b). Furthermore, the main characteristic diffraction peaks of Au NPs were observed for Au/ZC at 2θ values of 38.16 and 44.38°, corresponding to the (111) and (200) crystal planes of the face-centered cubic (FCC) structure of metallic Au, respectively (JCPDS no. 002-1095).

To substantiate the presence of Au NPs on the resulting Au/ZC, SEM–EDX analysis was carried out. As illustrated in Figure 9c, a uniform distribution of Au NPs on the surface of the ZC sample was observed. By employing high-resolution transmission electron microscopy (HRTEM) analysis, the deposited Au NPs exhibited a semispherical morphology with a well-defined metal/oxide interface (Figure 9d). Regarding the chemical composition and electronic state of Au/ZC, the survey scan illustrated the presence of Zn, Au, and O elements in the absence of impurities (figure not shown). The Zn 2p high-resolution XPS spectra of Au/ZnO displayed two asymmetric broad peaks during the binding at 1014–1023 and 1038–1046 eV (Figure 9e). After deconvolution, two subpeaks with binding energies of 1020.97 and 1044.15 eV corresponded to the spin orbits of Zn 2p_3/2_ and Zn 2p_1/2_ of Zn^2+^, respectively, while another two subpeaks located at 1017.89 and 1041.15 eV related to the spin orbits of Zn 2p_3/2_ and Zn 2p_1/2_ of non-lattice zinc ions, respectively. The high-resolution Au 4f XPS spectra of Au/ZnO displayed a broad peak, maximized at a binding energy of 88.07 eV (Figure 9f). Subsequent deconvolution revealed four distinct peaks corresponding to Au 4f_7/2_ (84.63 eV), Au 4f_5/2_ (87.10 eV), Zn 3p_3/2_ (88.59 eV), and Zn 3p_1/2_ (91.01 eV), thus confirming the existence of metallic gold on the ZnO surface.^89,90^

Regarding the light absorption capacity, the Au/ZC composite exhibited an absorption spectrum spanning the UV light region (λ < 390 nm) and visible light region, with a pronounced peak centered at 564 nm (Figure 9g). The UV light absorption of the Au/ZC sample originated from the absorption band edge of the ZnO nanostructure, while the absorption in the visible light region arose from surface plasmon resonance (LSPR) of Au NPs.^91−93^ Concerning the recombination rate, the resulting Au/ZC sample exhibited a considerably reduced PL emission in comparison with that of the original ZC sample (Figure 9h). This outcome can be ascribed to the intrinsic property of metallic Au NPs, which act as efficient electron trappers, facilitating the unrestricted movement of electrons along their surface and/or between the CB and their Fermi level.^94,95^ This characteristic enhances the separation efficiency of the electron–hole pairs.

**Figure 10:**
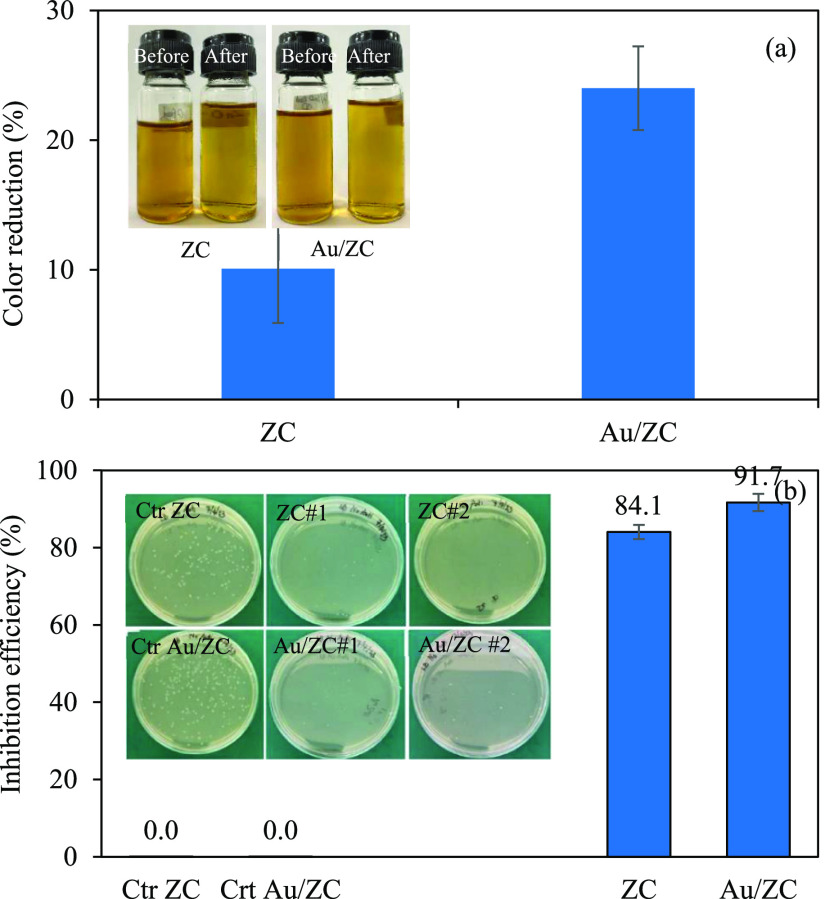
Photocatalytic activity of the resultant Au/ZC sample for (a) color reduction and (b) antibacterial activity.

To assess the potential photocatalytic applications of the resultant Au/ZC sample, two distinct photocatalytic processes were carried out, including the color reduction in distillery wastewater and antibacterial activity. Basically, upon exposure to incident light with photon energy equal to or higher than the bandgap energy, the ground-state electrons within the composite will be excited to CB, leaving the photogenerated holes at VB. These photogenerated holes serve as a strong oxidizing agent, which can oxidize H_2_O to form H^+^, O_2_, and OH^•^.^96^ The produced H^+^ can readily react with the photogenerated electrons to form H_2_.^16,97^ Besides, the photogenerated electron can react with dissolved O_2_, yielding super oxide radicals (O_2_^•–^) and hydrogen peroxide (H_2_O_2_).^98−101^ Based on the high oxidizing power of all formed reactive oxygen species (ROS): OH^•^, O_2_^•–^, and H_2_O_2_, they can oxidize some dissolved organic species in wastewater, initiating a cascade that leads to the formation of intermediates with reduced molecular weight and eventual CO_2_ production through complete oxidation.^96^ Moreover, it is believed that these generated ROS are known to inflict damage upon DNA, disrupt cell membranes, and degrade cellular proteins, ultimately resulting in cell death.^102−104^ As illustrated in Figure 10, it is evident that the Au/ZC sample outperformed the pristine ZC sample in terms of photocatalytic activity for both applications. Namely, the Au/ZC sample achieved a 24% increase in color reduction, approximately 2.4 times higher than that of the pristine ZC sample (Figure 10a). Additionally, the Au/ZC composite possessed an inhibition efficiency of around 97.1%, approximately 1.16 times higher than that of the pristine ZC sample (Figure 10b). The enhanced photocatalytic activity of the Au/ZC sample relative to that of the pristine one might be attributed to the presence of Au NPs on the ZC surface, which can enhance effective light harvesting as well as suppress the electron–hole recombination and thereby subsequently promote the photocatalytic activity. The effect of parameters and optimum condition of the color reduction and antibacterial activity were not further explored in this study as they were out of scope. Nevertheless, the outcomes presented here underscore the potential benefits and avenues for industrial waste remediation and utilization.

## 3 Conclusions

The present study delved into the comparative investigation of the photocatalytic recovery of gold ions from the gold-cyanide-contained wastewater. This exploration involved both commercial and hydrothermally synthesized ZnO NPs. Evidently, the commercial ZnO NPs exhibited superior photocatalytic activity in comparison to their synthesized counterparts. This disparity in performance can be attributed to the commendable crystal quality and appropriate defect content. The addition of suitable C_2_H_5_OH concentrations notably promoted the photocatalytic gold recovery from industrial gold plating effluent due to the synergetic interplay between a low oxidation potential and the resultant nonadsorbed products. A higher solution pH emerged as a driving factor for encouraging photocatalytic gold recovery. This outcome stems from the effective separation of electron–hole pairs facilitated by the elevated pH levels. The optimum condition for gold recovery was found to be at an initial wastewater pH of 11.0 with 10 vol % C_2_H_5_OH and 1.0 g/L of photocatalyst loading, in which nearly complete gold ion recovery was achieved within 7 h. In terms of the photocatalytic application, the Au/ZC sample exhibited a higher activity than the pristine ZC NPs for both color reduction and also the antibacterial ability due to the presence of decorated Au NPs on the ZnO nanostructure, which can accelerate the light absorption ability as well as the electron–hole separation efficiency.

## 4 Experimental Section

### 4.1 Preparation and Characterization of ZnO NPs

A facile hydrothermal synthesis method, adopted from Mohan et al.,^40^ was employed to synthesize the ZnO NPs. In brief, approximately 4.46 g of zinc nitrate 6-hydrate (Zn(NO_3_)_2_·6H_2_O; KemAus) was dispersed in 30 mL of distilled (DI) water. The resultant solution was thoroughly stirred at 600 rpm and 25 °C for 30 min. Next, the pH of the solution was adjusted to ∼12 by the dropwise addition of 5 M sodium hydroxide (NaOH, Merck). The solution was then transferred to a Teflon-lined stainless-steel autoclave and thermally treated at 125 °C for 2 h. At the designated time, the autoclave was allowed to cool naturally. The resultant solid portion was separated from the liquid mixture by centrifugation, followed by being gently washed with ethanol (C_2_H_5_OH, QRëC) 3 times and finally with DI water until the pH of the filtrate was equal to the pH of pure DI water. The ready-to-use ZnO NPs were obtained after drying at 80 °C for 4 h. A similar procedure was repeated by changing the autoclave temperature to 150 and 175 °C with a fixed time of 2 h. The commercial ZnO NPs and hydrothermally synthesized ZnO NPs at 125, 150, and 175 °C were denoted as ZC, Z125, Z150, and Z175, respectively.

The nanostructures of all explored ZnO NPs were observed by SEM (FE-SEM, JSM7610FPlus, JEOL). The crystallite structure and average crystallite were determined by an X-ray diffractometer (Bruker D2 Phaser) using Cu Kα X-ray. The surface area and porosity were measured via N_2_ adsorption/desorption at 77 K according to the Brunauer–Emmett–Teller (BET) methods via a gas adsorption analyzer (Quantachrome ASiQwin) using a degas temperature of 150 °C for 14 h in a N_2_ atmosphere. The optical property was examined by a *UV*–*vis* spectrophotometer (UV-1800, Shimadzu) and a luminescence spectrometer (PerkinElmer LS-55). The surface composition of all ZnO NPs was explored via XPS (Kratos Axis Supra+). The size of deposited Au was monitored by HRTEM (JEM-3100F, JEOL) using an accelerating voltage of 300 kV. The qualitative crystal defect was recorded by electron paramagnetic resonance spectroscopy (EPR, model, EMXmicro, Bruker) at 298 K.

### 4.2 Photocatalytic Activity

The photocatalytic activity of synthesized ZnO NPs was examined for gold recovery from cyanide-based gold plating wastewater. This effluent was simulated by mixing an actual plating bath solution taken from the circuit board industry in Thailand with DI water to get a concentration of gold ions of around 7–10 mg/L. In each experiment, approximately 300 mL of cyanide-based gold plating effluent with 0–20 vol % of selected hole scavengers (CH_3_OH, QRëC; C_2_H_5_OH, QRëC; C_3_H_7_OH, and QRëC) was utilized, and its temperature was roughly controlled at around 28–32 °C using the water circulation system equipped with the magnetic drive pump (PMD-0311, Sanso). Prior to the photoreaction, the dark experiment was carried out during the first 30 min in order to enhance a uniform dispersion of ZnO NPs in effluent as well as to promote the good adsorption of gold-cyanide species on the surface of photocatalysts. Afterward, the photocatalytic experiment was carried out using a 400 W high-pressure mercury lamp (PUV 533 BC) with a power density of 4.93 mW/cm^2^. At a particular time, approximately 5 mL of the processed effluent was collected and subjected to analysis of the gold ion concentration by flame atomic absorption spectrometry (Flame-AAS, Analyst 200+ flas 400; PerkinElmer). The percentage of gold recovery was calculated according to eq 12.
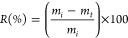
12where *R* is the gold recovery percentage and *m*_i_ and *m*_t_ are the initial and final mass of gold ions, respectively.

Besides, the kinetic rate of gold recovery was fitted with the pseudo-first-order reaction according to the Langmuir–Hinshelwood model (eq 13^105^). A plot of ln(*C*_t_/*C*_0_) against *t* gives the negative slope, which allows to determine the reaction rate constant.
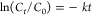
13where *C*_0_ is the initial concentration of gold ions, *C*_t_ is the concentration of gold ions at time *t*, and *k* is the pseudo-first-order rate constant.

### 4.3 Possible Applications of Resultant Gold-Decorated ZnO NPs

The photocatalytic activity of the resultant gold-decorated ZnO (Au/ZC) NPs was preliminarily tested via two applications: color reduction and antibacterial activity. For the color reduction, approximately 0.3 g of Au/ZC sample was interspersed in 100 mL of a 100-time diluted distillery slope taken from the alcohol production plant. To obtain uniform dispersion and adsorption of active species on the photocatalyst surface, the mixture was constantly stirred at 300 rpm in the absence of irradiated light. Then, the system was irradiated with a 400 W high-pressure mercury lamp (PUV 533 BC) at a power intensity of 4.50 mW/cm^2^ for 4 h. Afterward, the remining wastewater was taken and separated from the solid powder by centrifugation at 11,000 rpm (Eppendorf, 5804R) for 15 min, and subsequently, the light absorbance was measured at 453 nm using a *UV*–*vis* spectrophotometer (UV-1800, Shimadzu). The color reduction efficiency was computed according to eq 14.
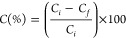
14where *C* is the color reduction percentage and *C*_i_ and *C*_f_ are the initial and final colors of the wastewater in the Pt–Co unit, respectively.

The photocatalytic antibacterial activity was examined via the growth inhibition efficiency of *Escherichia coli* (*E. coli*) DH5α. Initially, *E. coli* was cultured in Luria–Bertani (LB) medium at 37 °C in the incubator shaker overnight. The density of the cell was measured spectrophotometrically at an optical density of 600 nm. The starting cell optical density of 1 was then further 10-fold serially diluted to 1 × 10^–5^ with LB broth. Approximately 900 μL of prepared bacterial solution was transferred into a glass tube. About 100 μL of a well-dispersed catalyst solution at 100 mg/mL of catalyst concentration was added to cell solution. Then, the glass tube was irradiated with a 400 W high-pressure mercury lamp (RUV 533 BC) at a power intensity of 4.50 mW/cm^2^ for 4 h. For the whole experiment, the system temperature was roughly controlled at around 28–32 °C by the water circulation system driven by a magnet drive pump (PMD-0311, Sanso). Afterward, 200 μL of solution was taken and transferred to an LB agar plate for incubation at 37 °C overnight. The presence of colony-forming units (CFUs) was counted and compared with that in the absence of a photocatalyst sample. The inhibition efficiency (η) was calculated from the difference of colony numbers in the absence and presence of photocatalysts (eq 15).^102^
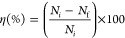
15where *N*_i_ and *N*_f_ are the number of colonies on the plates in the absence and presence of photocatalysts.
